# PLGA-Quercetin Nano-Formulation Inhibits Cancer Progression via Mitochondrial Dependent Caspase-3,7 and Independent FoxO1 Activation with Concomitant PI3K/AKT Suppression

**DOI:** 10.3390/pharmaceutics14071326

**Published:** 2022-06-23

**Authors:** Neera Yadav, Amit Kumar Tripathi, Amna Parveen, Shama Parveen, Monisha Banerjee

**Affiliations:** 1 College of Pharmacy, Gachon University, #191, Hambakmoeiro, Yeonsu-gu, Incheon 21936, Korea; yadavneera21@gachon.ac.kr; 2 Molecular and Human Genetics Lab, Department of Zoology, University of Lucknow, Lucknow 226007, India; shamaparveen1192@gmail.com; 3 Electrophysiology Lab, School of Biomedical Engineering, Banaras Hindu University, Varanasi 221005, India; amitbt2008@gmail.com

**Keywords:** quercetin, anti-cancer, phytochemical, drug delivery, FoxO1

## Abstract

Quercetin is one of the most important plant flavanols, having several pharmacological and biological uses. Quercetin (Q) is an extremely hydrophobic phytochemical and has poor intracellular absorption, which makes its use limited. Present research demonstrates that quercetin-loaded PLGA nanoparticles (PLGA-QNPs) could overcome its low hydrophilicity and improve its anti-cancer potential. PLGA nanoparticles loaded with Q were prepared by the solvent evaporation technique and its anticancer activity was examined in vitro as well as in vivo. The cell viability was assessed through MTT assay and apoptosis was assayed through Hoechst-PI and EB/AO double staining followed by mitochondrial damage through Mito-tracker RMX-Ros. Gene expression was examined through RT-PCR. Cell cycle arrest in G2/M phase was analyzed through FACS. The results obtained revealed that PLGA-QNPs significantly reduced the viability of human cervical and breast cancer cell lines. PLGA-QNPs induced apoptosis in human cervical cancer cells in a dose dependent manner. The gene expression of PI3K/AKT was down-regulated and FoxO1 was upregulated in PLGA-QNP-treated cells, which showed a high expression level of active Caspase-3 and 7, which are responsible for apoptosis. In addition, PLGA-QNPs reduced the average number of tumors and prolonged the tumor latency period in DMBA-induced mammary adenocarcinoma SD rats. These findings suggest that PLGA-QNPs inhibit cervical and breast cancer progression via mitochondrial dependent Caspase-3 and 7 and mitochondrial independent FoxO1 activation with concomitant suppression of the PI3K/AKT pathway. For future studies, we suggest that potential druggability efficacy and clinical development of anticancer PLGA-QNPs need to be evaluated intensely for successful anticancer drug development.

## 1. Introduction

Cervical cancer is the fourth leading cause of death worldwide, and ranks third among cancer cases in women. Cervical cancer incidences are expected to be augmented by 70% in the next 2 decades [1]. It is estimated that each year approximately 500,000 new cases of cervical cancer are identified with >250,000 deaths globally. The main causative agent of cervical cancer is oncogenic types of human papilloma virus (HPV). Effective ways to treat cervical cancer should be introduced because it is a global cause of mortality and morbidity in women. Major treatment methods include chemotherapy, radiotherapy and surgery. In some cases, combined treatment methods are required to treat patients in different stages. Chemotherapy and radiotherapy exert innumerable side effects, especially damage to other non-targeted tissues, hair loss, anemia, nausea, multidrug resistance, and in some cases neurotoxicity [2]. The chances of recurrence are about 35% and most patients eventually die. Hence, the development of novel anticancer drugs with enhanced effectiveness and reduced side effects is indispensable. Interestingly, phytochemicals have been recognized as unsurpassed complementary and alternative medicines with or without chemotherapy and/or radiotherapy [3]. A number of phytochemicals and their derivatives with reduced side effects have been reported to have enhanced apoptosis and decreased cell proliferation activity [4].

Quercetin, a known phytochemical has diverse biological actions including antioxidant properties, anti-inflammatory, antimicrobial, and anticancer activities [5]. Quercetin is also useful in treating diabetes, bladder infections, arthritis, and heart problems. It has been reported to have an anti-proliferative effect on human ovarian, stomach, and breast cancer MCF-7 cells [6]. Despite its valuable biological properties, the main drawback of Q is that it is insoluble in water, which impedes its use as an effective treatment against several diseases [7]. Quercetin is enormously hydrophobic in nature and its usage is restricted by its poor absorption and retention in the cell. However, alternative strategies such as preparing nanoformulations or a combination of phytochemicals with nanoparticles can improve overall water solubility, bioavailability, and targeted delivery at the tumor site, which in turn enhances the therapeutic capability of phytochemicals [8]. For example, previous studies indicate that quercetin nanoparticle co-treatment with cisplatin downregulates Wnt16 expression in a bladder carcinoma model [9]. In addition, QNPs have been reported for their capability to remodel the tumor microenvironment and improve the effect on kidney disease, as well as restrict inflammatory cell infiltration by downregulating intercellular adhesion molecular-1 (ICAM-1) expression [10]. Lou et al. reported downregulation of PI3K/AKT and Bcl-2 by QNPs in human neuroglioma cells. QNPs also modulated LC3 and ERK, cytoplasmic p53, cleaved Caspase-3, PARP and p-mTOR expression [11]. QNPs inhibited cervical cancer proliferation and increased apoptosis by inducing JAK2 suppression in a BALB/c nu/nu nude mice model [12]. QNPs decreased the expression of Bcl-2 with concomitant increased Bax expression in MCF-7 breast cancer cells [13]. Furthermore, Q acts as an inhibitor of breast cancer resistance protein (BCRP) in SD rats [14]. In another study, quercetin-encapsulated chitosan-activated copper oxide nanoparticles decreased the proliferation of mammary carcinoma in breast cancer rat models [15]. PLGA or poly(DL-lactide-co-glycolic acid) is a biodegradable polymer that has high stability, biocompatibility, and biodegradability in cellular environments. It is usually degraded into individual monomers by hydrolysis of its ester linkages and then metabolized and removed by natural functioning of the cell. 

In this study, we propose that PLGA-QNPs, a representative Forkhead box protein O1 (FoxO1)-activating nanoformulation, are a potential candidate against cervical and breast cancer. The intention of using PLGA-QNPs is to increase the bioavailability within the cell as well as slow down release of quercetin into the blood stream, thereby enhancing its overall therapeutic activity. Our study examines the underlying pathway that can serve as a target for PLGA-quercetin nanoformulations to treat different types of cancer. However, the potential druggability efficacy and development of anticancer PLGA-QNPs need to be investigated extensively at the clinical level in the near future. 

## 2. Materials and Methods

### 2.1. Materials

Quercetin (Mol. wt. 302.236 gm/mol), polyvinyl alcohol (PVA), Rhodamine 123, Trypan blue, 0.25% Trypsin-EDTA solution, Fetal bovine serum (FBS), Antibiotic-antimycotic (Ab/Am) solution, 3-(4,5-dimethylthiazol-2-yl)-2,5-diphenyltetrazolium bromide tetrazolium salt (MTT), Paraformaldehyde, Minimal essential medium (DMEM F-12 HAM), Caspase-3 kit, Syber green (for RT-PCR), Acridine orange (AO), Acetone, Ethidium bromide (EB), Bisbenzimide/Hoechst 33342 (Himedia, Mumbai, India), Methanol, Mitotracker red CMX-Ros, Propedium iodide (PI), Phosphate burred saline (PBS), Vybrant ^®^ FAM Caspase-3 and -7 Assay Kit (Invitrogen, Waltham, MA, USA), dimethylsulphoxide (DMSO), 50:50 Poly (D,L-lactideco-glycolide) (PLGA), Dichloromethane (DCM) were purchased from Sigma Chemical Co. (St. Louis, MO, USA). Cell culture flasks (25 and 75 cm^2^), petri dishes (35 and 90 mm), multi well microplates (6 and 96 wells), cell scraper, and cryovials were procured from M/s Nunc, Waltham, MA, USA. Centrifuge tubes (15 and 50 mL), microcentrifuge tubes (0.5–2.0 mL) were purchased from M/s Tarsons India Ltd. (Kolkata, India). Syringe, filters and filter membranes were procured from M/s Millipore India Ltd. (Bengaluru, India).

### 2.2. Synthesis and Characterization of PLGA-QNPs

Q nanoparticles were prepared by the solvent evaporation method with slight modification. The ratio of Q and polymer was kept 1:10 for nanoparticle synthesis. Beforehand, 10 mg Q was dissolved in 2 mL acetone and 100 mg PLGA was dissolved in 2 mL DCM. Q was then added to DCM-dissolved PLGA to make the oil phase. The oil phase was added to aqueous PVA (5% in MQ). Subsequently, the solution was sonicated for 10 min on ice bath. The mixture was stirred for evaporation of organic solvent for about 7 h. The formed nanoparticles were then washed with deionized water thrice to eliminate unreacted drug and residual PVA. Further, the nanoparticles were ultracentrifuged at 36,000 rpm for 40 min. The obtained powder was then lyophilized to collect pure nanoparticles.

The nanoformulation was characterized by different methods such as drug loading, entrapment efficiency, Fourier Transform Infrared Spectroscopy (FTIR), Zeta potential and Scanning Electron Micrograph (SEM). 

### 2.3. Drug Loading and Encapsulation Efficiency (EE)

Synthesized nanoparticles were analyzed spectrophotometrically to determine the drug loading and encapsulation efficiency by using a Lambda 35 UV/VIS Spectrophotometer (Perkin Elmer, Waltham, MA, USA). First, 10 mg of QNPs was dissolved in 10 mL of DCM + acetone (50:50 *v*/*v*) and then absorbance was recorded at 242 nm. The amount of quercetin present in the nanoformulation was calculated in the form of encapsulation efficiency (%EE) and the percentage loading (%DL) using the following formula: %EE = (Amount of quercetin in entrapment − amount of quercetin in supernatant)/(Total amount of quercetin in formulation) × 100

%DL = (weight of quercetin in formulation × 100)/(Weight of formulation)

### 2.4. Fourier Transform Infrared Spectroscopy 

FT-IR spectra were recorded on a Perkin Elmer FTIR Spectrometer on a KBr plate (Perkin Elmer, USA) (scan range: 4000–500 cm^−1^, resolution: 4.0 cm^–1^, number of scans: 16). ^1^HNMR was recorded on a Bruker DRX-300 spectrometer (Bruker, Berlin, Germany) using appropriate solvents. 

### 2.5. Determination of Particle Size and Charge 

The average particle size distribution and zeta potential of the QNPs were measured by using dynamic light scattering (DLS) on Zetasizer-Nano-ZS (Malvern, UK). 

### 2.6. Surface Morphology of QNPs

The surface morphology of QNPs was analyzed by a scanning electron microscope equipped with TEAM EDS System (EDAX, Mahwah, NJ, USA). First, the sample was prepared by coating the nanoparticles with gold film and images were captured while maintaining high vacuum conditions.

### 2.7. Cell Culture and Treatment Conditions

The human cervical cancer cell line (HeLa) and breast cancer cell line (MCF-7) were procured from the National Centre for Cell Sciences, Pune, India and maintained in a CO_2_ incubator at 37 °C, 5% CO_2_ and 95% relative humidity. DMEM F-12 HAM supplemented with 10% FBS and 1.5% antibiotic-antimycotic solution was used as cell growth medium. Cells up to six passages and cultured for less than 4 weeks were used in all experiments. Cells were grown up to 70–80% confluence and treated with clinically safe concentrations (1, 10, 25 and 50 µM) of QNPs and bulk quercetin and incubated for different time intervals as per the requirement of the individual experiment.

### 2.8. Cell Morphology Study

Cells were grown in 35 mm culture plates at a confluence of 70–80%. Different concentrations (1, 10, 25 and 50 µM) of QNPs and bulk quercetin were prepared in fresh culture medium. Cells were treated with the formulation and incubated for 48 h. Control samples were also run parallel. After incubation, cells were washed with PBS to remove dead cells, observed under an inverted microscope and images were captured at 20× magnification (Nikon, Tokyo, Japan).

### 2.9. MTT Assay

The assay is based on the ability of the cells to convert the yellow 3-(4,5- dimethylthiazol-2-yl)-2,5-diphenyltetrazolium bromide salt into a purple MTT formazan, by mitochondrial dehydrogenase enzyme, which was measured spectrophotometrically. The mitochondrial integrity and activity is interpreted as a measure of cell viability. In brief, after treatment with the formulation, the reaction mixture was carefully taken out and the MTT solution of 5 mg/mL was added at 1:10 with media and incubated for 4 h in a CO_2_ incubator. After incubation, the culture media was discarded and crystals of formazan were dissolved in DMSO by keeping them on a shaker for 20 min. The color of the dye was read at 530 nm [16].

### 2.10. Hoechst-PI Double Staining 

Cells were grown in 35 mm culture plates and treated with different concentrations of QNPs as described earlier. Cells were washed with cold PBS and 10 µL of Hoechst dye mixed in 500 µL PBS was added and incubated for 10 min at RT. Cells were then washed with PBS and 5 µL PI mixed in 500 µL PBS was added to the cells and incubated for the next 10 min at RT. Cells were then observed under fluorescence microscope and images were captured.

### 2.11. EB/AO Double Staining 

The EB/AO assay is based on the binding of vital dye to the condensed and fragmented DNA during the process of apoptosis, whereas the inability to exclude vital dye results in cell necrosis, leading to orange staining of nuclei. This assay is specifically used for qualitative analysis and differentiation between live, apoptotic, and necrotic cells. Once QNPs and Q treatment to the cells was over, cells were incubated in fresh culture medium for 2–18 h. A cocktail of EB and AO (100 µg/mL each) was prepared in PBS. To each well, 8 µL cocktail was added. After 10 min incubation, cells were observed under fluorescence microscope [17].

### 2.12. Mito-Tracker Staining

Treated cells were incubated with Mito-tracker RMX-Ros (100 nM) for 15 min at 37 °C. Cells were washed with pre-warmed PBS/DMEM twice and fixed with 4% paraformaldehyde. Cells were then permeabilized with 0.2% triton X-100 for 20 min at 37 °C and washed with PBS thrice. Finally, images were acquired by inverted fluorescence microscopy [18].

### 2.13. Caspase 3 and 7 Measurements

Caspase 3 and 7 was measured using the Vybrant ^®^ FAM Caspase 3 and 7 Assay Kit. The methodology consists of fluorescent inhibitor of caspases (FLICA™)-based detection of active caspases. The cells were incubated for 24 and 48 h with concentrations of 1 µM and 10 µM. The selection of doses was based on the results obtained from MTT and NRU assay. The procedure was conducted as per the manufacturer protocol [19].

### 2.14. Cell Cycle Arrest

Cells were seeded in 6 well plates for 24 h to grow and then treated with QNPs (1 and 10 µM) at 70–80% confluence. The dose selection was based on the MTT and NRU measurements as well as physiological condition of the cells. Treated cells were carefully washed twice with PBS and incubated in fresh culture medium for 2–6 h. After incubation, cells were harvested with trypsin and suspended in fresh culture medium. Cells were centrifuged at 1000 rpm for 5 min, and the pellet was suspended in 1 mL PBS followed by 2.5 mL of 70% ethanol. Suspension was incubated on ice for 15 min or kept at −20 °C overnight. Cells were again centrifuged at 1500 rpm for 5 min. The pellet was suspended in 500 µL PI solution (50 µL/mL PI + 0.1 mg/mL RNase-A + 0.05% Triton X-100) and incubated at RT for 40 min. PBS (3 mL) was added to the suspension and then centrifuged at 1500 rpm for 5 min. The supernatant was removed carefully to avoid loss of cells. Finally, the pellet was suspended in 500 µL PBS and analyzed for cell cycle arrest through FACS (FACS Caliber BD) using the Cell Quest program and Mod Fit software (Version 3.3, Lucknow, India) [20].

### 2.15. Real-Time PCR

After treatment with different concentrations of QNPs (1, 10 and 25 µM, based on the physiological condition of the cells) and incubation for 24 h, total RNA was isolated by using TRIzol reagent as per the manufacturer’s protocol (Life technologies). After quantification of RNA at 260 nm by Nano-drop spectrophotometer (ND-1000 Thermo scientific), cDNA was synthesized by using a high-capacity cDNA Reverse Transcription Kit. The relative expression of p-Akt, PI3K and FoxO1genes (each sample in triplicate) was carried out with Real-Time PCR (Applied Biosystems-7900 HT Fast-Real-Time PCR system) using ABI–sequence detection system (PE Applied Biosystems, Foster City, CA, USA). The various steps of RT-PCR comprise the initial denaturation for 10 min at 95 °C, 40 cycles of 95 °C for 15 s and 50 °C for 60 s. The 2^ΔΔct^ method was used to calculate the fold change in the expression of genes where the cycle threshold or CT values were normalized with the housekeeping gene b actin [16].

### 2.16. Effect of PLGA-QNPs on DMBA Induced Mammary Adenocarcinoma SD Rats

All experiments and surgical procedures were conducted as per the protocol for animal use and approved by the Central Animal Ethical Committee at IMS, BHU, Varanasi. Inbred Female Virgin SD rats (220 ± 30 g) were acclimatized for two weeks under conditions of 12-h light/dark cycle, constant humidity and controlled temperature (25 ± 2 °C). Water and standard pelleted diet were provided ad libitum. Animals were starved of food for 8–10 h with free access to water before the experiment [21].

Virgin female SD rats (42–45 days old) were divided into 5 groups of 6 animals each. Group 1 comprised untreated normal animals, Group 2 served as a positive control where animals received only DMBA treatment, Group 3 includes the intraperitoneal (i.p) injection of Q (100 mg/kg), Group 4 consists of rats treated with PLGA dissolved in acetone and Group 5 animals received QNP (128 mg/kg, body weight, i.p) treatment in addition to the DMBA dosage. 

The rats were fed with 7 mg DMBA per rat (Sigma-Aldrich; St. Louis, MO, USA) in olive oil, orally by gavage, once a week, for 3 weeks. The rats were palpated for mammary tumors once a week, starting from the fourth week after administration of DMBA. Average number of tumors per tumor-bearing rat, percentage of animals with tumor and tumor latency period were recorded for a period of 7 weeks (Bansal et al., 1970). The animals were sacrificed under anesthesia, and the tumors were extricated and weighed. Percentage of tumor inhibition was calculated by the formula (1–B/A) × 100, where ‘A’ is the average tumor weight of the control group and ‘B’ is that of the treated group.

### 2.17. Statistical Analysis

Each experiment was repeated at least three to four times in duplicates. Data were articulated as mean ± SE and evaluated by one-way ANOVA and Tukey’s post hoc test. *** *p* < 0.001, ** *p* < 0.01 and * *p* < 0.05 were considered statistically significant.

## 3. Results

### 3.1. Characterization of QNP Formulation

#### 3.1.1. The Particle Size of QNPs Was Suitable for Nanoformulation 

**Figure 1:**
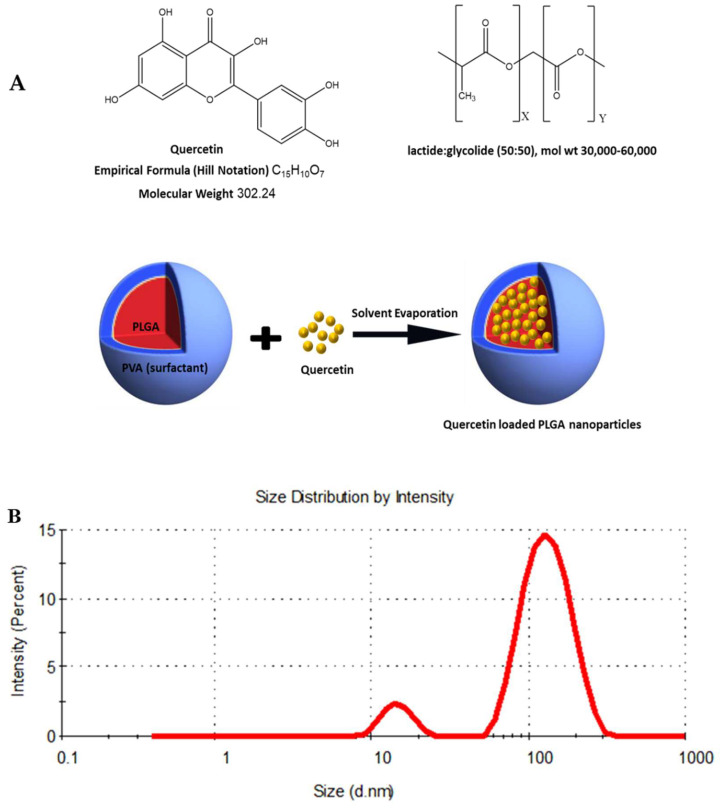

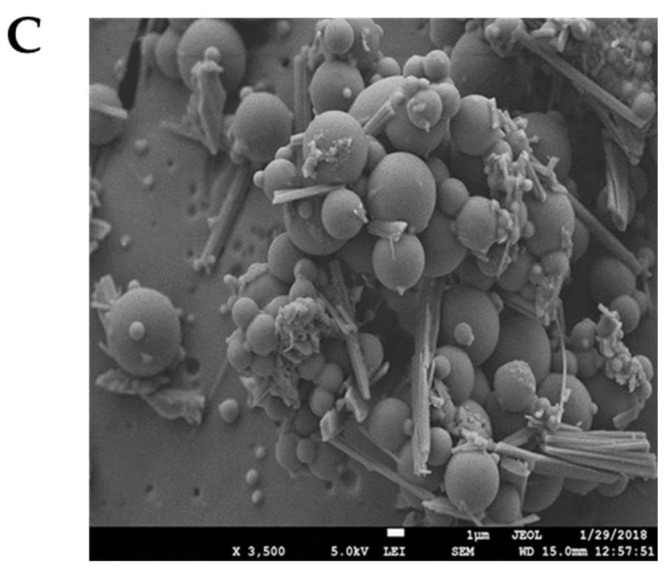
(**A**) The structure of quercetin, PLGA and Schematic representation of the technique for preparation of the quercetin-PLGA nanoparticle formulation. (**B**) DLS size distribution of the Q-PLGA. (**C**) Scanning electron microscopy (SEM) microphotographs of Q-PLGA. Scale bars: 100 nm.

QNP formulations exhibited a %EE, zeta potential, and PDI of 63.2%, −19.8 mV, and 0.3 ± 0.03 respectively. The average particle size measured by SEM was 89.8 ± 5.9 nm. The particles of the QNP nanoformulation had sufficient repulsion, which was capable of avoiding particle aggregation for long term action (Figure 1).

#### 3.1.2. FT-IR Spectroscopy Confirmed Q Encapsulation within PLGA

**Figure 2:**
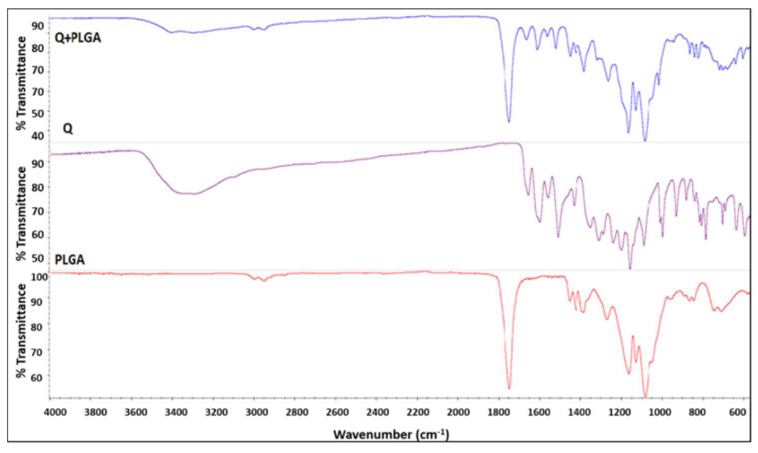
FT-IR spectra of Q-PLGA.

Spectra for free PLGA nanoparticles showed the characteristic bands of the polymer, –CH, –CH2, –CH3 stretching was observed in the range 3000–2900 cm^−1^, and the carbonyl –C=O and C–O stretching was observed in the range 1800–1700 cm^−1^ and 1300–1000 cm^−1^. Besides this, the bending stretching was observed at 1450–1350 cm^−1^ for –CH3. For quercetin, the spectra showed the characteristic bands corresponding to OH groups (3600–3000 cm^−1^), to C=O absorption (1650 cm^−1^), bands of C=C aromatic stretching (1650–1400 cm^−1^), C–H bending in the plane and out of plane (1050–950 cm^−1^) and (850–600 cm^−1^), a band attributed to the C–O stretching of the oxygen in the ring (1300–1250 cm^−1^), and the region for C–O aromatic stretching (1250–1150 cm^−1^). For quercetin-loaded PLGA nanoparticles, spectra showed that the OH stretching band (3200–3600 cm^−1^) is slightly shifted and increased in terms of energy absorption. These observations suggest that quercetin is associated with the PLGA polymer by hydrogen bonds. In addition, in the quercetin-loaded PLGA nanoparticle, the band corresponding to C=O stretching (1800–1700 cm^−1^) was broader, indicating that quercetin is associated with the PLGA polymer by interactions between the carbonyl and the carboxyl groups of the quercetin and the polymer. In other words, IR measurements confirmed the encapsulation of quercetin within the PLGA to form nanoparticles (Figure 2).

### 3.2. PLGA-QNPs Induced Cell Morphology Destruction in Cervical Cancer Cells

**Figure 3:**
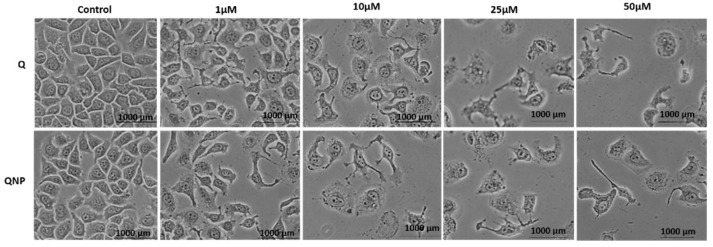
Phase contrast microscopy of the HeLa cell line treated with different concentrations of Q or QNPs for 48 h. The normal morphology of cells was lost at higher conc.

It was observed that QNPs were able to alter the surface morphology of cells unfavorably when treated for 48 h. The morphological changes observed were gradual with increasing concentration. Untreated cells appeared healthy after 48 h, while treated samples showed surface destruction in the following order: 50 µM > 25 µM > 10 µM > 1 µM. QNPs exhibited a more destructive response in surface morphology maintenance compared to Q. The highest effect was observed in cells treated with 50 µM QNPs with a very few residual cells remaining (Figure 3).

### 3.3. PLGA-QNPs Reduced the Viability of Cervical and Breast Cancer Cell Lines

**Figure 4:**
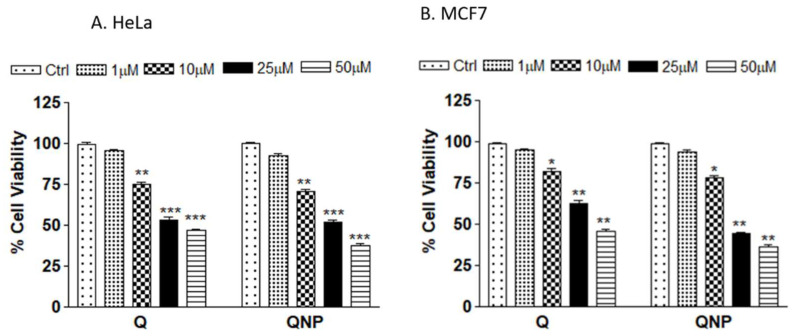
Percent viability of cancer cells treated with different concentrations of Q or QNPs for 24 h, (**A**) Human cervical cancer cell line HeLa; (**B**) MCF-7 breast cancer cell line. * *p* < 0.05 or ** *p* < 0.01 or *** *p* < 0.001 as compared to control.

Cell viability through MTT assay in the human cervical cancer cell line (HeLa) and breast cancer cell line (MCF-7) revealed that QNPs significantly inhibited viability of the cells. A gradual decrease in cell viability was observed with increasing conc in the following order: 50 µM > 25 µM > 10 µM > 1 µM. Similar results were obtained in MCF-7 cell lines. The % cell viability of QNP (1 µM, 10 µM, 25 µM and 50 µM)-treated HeLa and MCF-7 cells was 92.62 > 70.84 > 51.89 > 37.89 and 87.1 > 72.74 > 55.61 > 47.34, respectively. The % cell viability of Q-treated HeLa and MCF-7 cells was as follows: 95.47 > 75.34 > 54.65 > 45.89 and 95.93 > 79.88 > 63.25 > 51.72, respectively. The reduction in % viability was higher in QNP-treated cells, indicating that QNPs were more toxic than Q (Figure 4).

### 3.4. PLGA-QNPs Induced Apoptosis in Cervical Cancer Cell Lines 

**Figure 5:**
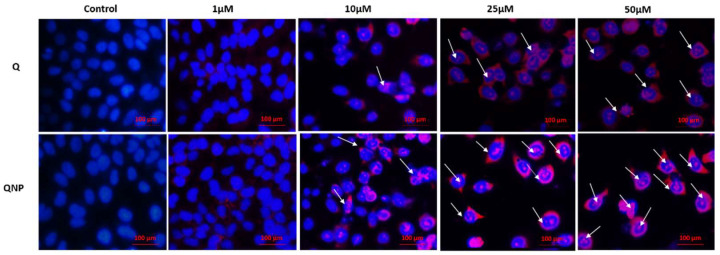
Fluorescence micrographs of Hoechst-PI double stained HeLa cell line treated with different concentrations of Q or QNPs for 48 h. Apoptotic cells are indicated by arrows.

Results obtained from Hoechst-PI double stain revealed that QNPs were able to induce apoptosis in cells. Untreated cells appeared blue due to Hoechst alone binding with the nuclear material. Cells treated with different conc. of quercetin or QNPs underwent apoptosis with increasing concentrations and demonstrated accrual of both dyes, i.e., Hoechst (blue) and PI (red) due to nuclear membrane damage in the following order: 50 µM > 25 µM > 10 µM > 1 µM. However, 1 µM-treated samples showed no significant changes and appeared like the control samples. Additionally, QNPs showed a greater apoptotic effect over Q for all concentrations (Figure 5).

### 3.5. PLGA-QNPs Induced Apoptosis and Necrosis in Cervical Cancer Cell Lines 

**Figure 6:**
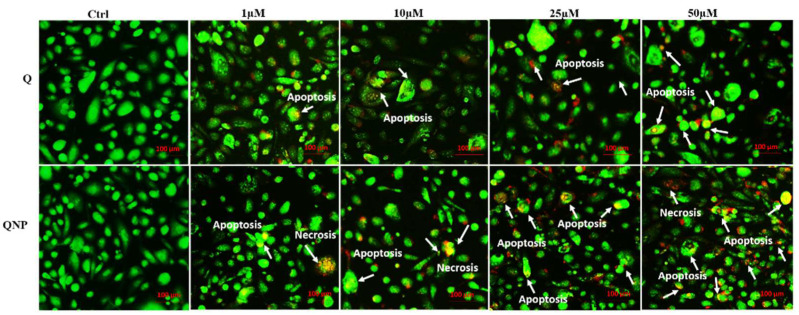
Fluorescence micrographs of EB/AO double stained HeLa cell line treated with different concentrations of Q or QNPs for 48 h. Apoptotic (light orange) and necrotic (dark orange) cells are indicated by arrows.

Further confirmation of apoptosis by EB/AO assay revealed the highest effect in 50 µM-treated cells. Whereas, untreated cells appeared healthy and stained green due to AO staining only. Nuclei of the cells with different fates appeared green (live and healthy), light-orange (apoptotic), or dark orange (necrotic). Higher QNP concentrations caused more membrane damage, which allowed cells to stain with both dyes, i.e., EB and AO, indicating more apoptosis and necrosis as compared to bulk quercetin (Figure 6).

### 3.6. PLGA-QNPs Induced Mitochondrial Damage in Cancer Cells

**Figure 7:**
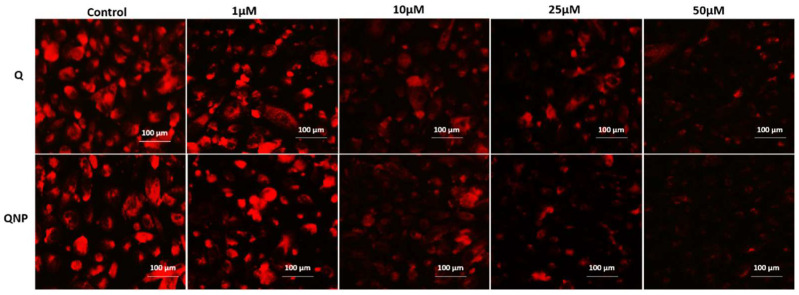
Fluorescence micrographs of Mito-tracker Red RMX-ros-stained HeLa cells treated with different concentrations of Q or QNPs for 48 h. Bright red color is indicative of live mitochondria.

For measurement of mitochondrial damage by QNPs, Mitotracker Red CMX-Ros was used. The effect of QNPs on mitochondrial damage was concentration dependent in the following order: 50 µM > 25 µM > 10 µM > 1 µM. Untreated cells stained consistently with the dye, whereas only traces stained for 50 µM conc.-treated cells due to extensive mitochondrial damage. A similar pattern was observed with Q-treated cells but the effect was to a lesser extent (Figure 7).

### 3.7. PLGA-QNPs Induced Caspase Activation in Cervical Cancer Cells

**Figure 8:**
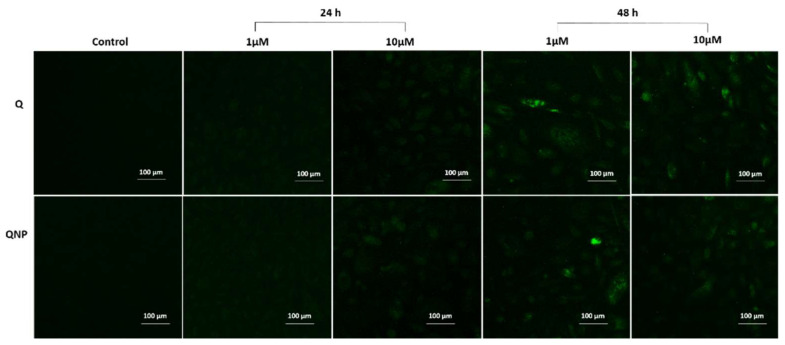
Fluorescence micrographs of FLICA caspase 3 and 7-stained HeLa cells treated with 1 µM and 10 µM concentrations of Q or QNPs for 24 and 48 h.

Caspase activity in the cells was measured in the form of active caspase 3 and 7, which specifically bind with a cell permeable fluorescent probe FLICA. The results revealed increased caspase activity after 48 h incubation of cells treated with 1 and 10 µM of QNPs. The active caspase measurement was both concentration and time dependent (Figure 8). 

### 3.8. PLGA-QNPs Arrested Cancer Cells in the G2 Phase of the Cell Cycle

**Figure 9:**
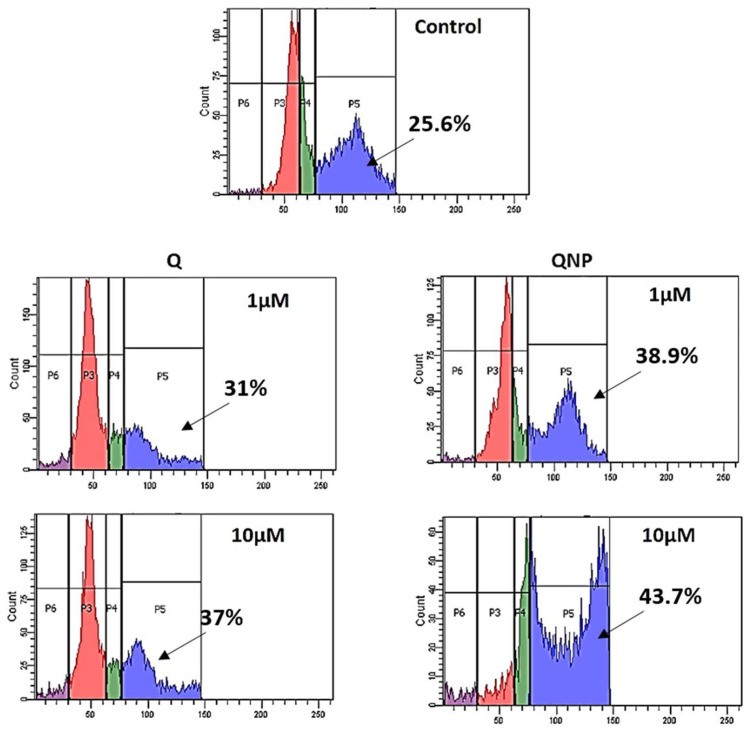
Cell cycle analysis of Q or QNP-treated HeLa cells. After Q or QNP treatment, cells were incubated in fresh medium for 2–6 h and analyzed through FACS. QNP (10 µM)-treated cells showed 43.7% G2 phase arrest.

Two concentrations (1 µM and 10 µM) each of QNPs and Q were used for cell cycle analysis, which demonstrates cell distribution in different phases of the cell cycle through flow cytometry. PI binds specifically with the DNA of cells in different phases. The result obtained from flow cytometry indicated that the number of cells in the G2 phase was greater in 10 µM QNP-treated cells followed by 1 µM QNPs, 10 µM Q and 1 µM Q. Untreated sample showed a normal distribution of cells in different phases (Figure 9).

### 3.9. PLGA-QNPs Triggered Downregulation of PI3K/p-Akt with Concomitant Upregulation of FoxO1 Genes

**Figure 10:**
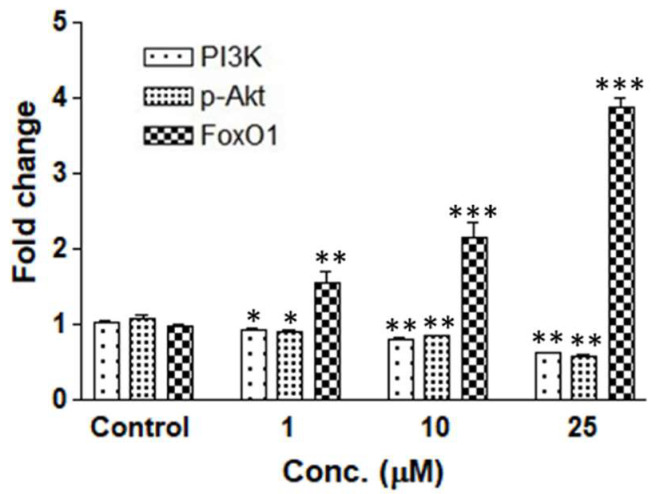
Fold change of PI3K, p-Akt and FoxO1 gene expression of QNP-treated HeLa cells. * *p* < 0.05 or ** *p* < 0.01 or *** *p* < 0.001 as compared to control.

QNP-induced expression of p-Akt, PI3K, and FoxO1 genes was quantified through real-time PCR. Downregulation of PI3K and p-Akt and upregulation of FoxO1 was found to be 0.6-fold, 0.59-fold and 3.87-fold, respectively, at 25 µM while concomitantly, in untreated cells, the expression was at the basal level (i.e., 1.0-fold). Each sample was normalized with b actin. b actin was used as an endogenous control in all samples, and it was found uniformly, which confirmed that mRNA maintained its integrity during the study (Figure 10).

### 3.10. PLGA-QNPs Reduced the Tumor Volume and Tumor Weight in Rats 

Table 1 shows the tumor volumes of control, vehicle treated by PBST, vehicle control treated by only PLGA, Q (100 mg/kg) and QNP (128 mg/kg). There was a significant increase in the tumor volume of mammary cancer rats treated with vehicle and PLGA, compared with drug treated rats, viz Q and QNP. Q (*p* < 0.05) and QNP (*p* < 0.05)-treated rats showed a significant reduction in tumor volume when compared with mammary tumor control rats and treatment with only PLGA. However, QNP post-treatment with 4 weeks followed by Q single dose exhibited a maximum reduction (*p* < 0.01) in tumor volume when compared with control and PLGA treated rats. In addition, body weight, tumor incidence and tumor weight were decreased in QNP-treated SD rats (Table 2).

## 4. Discussion

The burden of cervical and breast cancer cases can be reduced by implementing improved diagnostic technologies and medical treatment accessibility. However, more targeted approaches are required to make progress in these cancers. Nanotechnology such as the combination of phytochemicals with nanoparticles has been considered a more updated approach for the targeted delivery of anticancer drugs to enhance its therapeutic effects with reduced side effects.

Quercetin, a dietary flavonoid, comprising antioxidant, antiproliferative, anti-inflammatory, antiangiogenic, pro-apoptotic, and anti-cancerous properties has been considered as an attractive alternative compound to slow down progression of various cancers including cervical and breast cancer [22,23,24]. However, the hydrophobic nature of quercetin is responsible for its poor absorption and retention in the cell, which in turn affect its potential therapeutic effect [25]. Additionally, earlier studies have advocated better efficacy of nanoparticle based drug delivery systems that enhance bioavailability of the drugs in nano-formulations over the traditional drugs [26]. Therefore, an alternative approach has been designed to enhance the therapeutic activity of quercetin against cervical or breast cancer [27]. Synthesis of nanoparticles using natural products entrapped in natural polymers is now an updated technology for enhanced absorption of such problematic compounds [28]. In this study, Q nanoparticles were efficaciously synthesized using the solvent evaporation method with slight modification and its effectivity against cancer cells was studied through growth inhibition and enhanced cell death. Results indicated that quercetin is associated closely with the PLGA polymer by interactions between the carbonyl and the carboxyl groups of the quercetin and the polymer. Furthermore, the encapsulation of quercetin within the PLGA nanoparticle was confirmed with IR measurements that probably increased its cellular bioavailability. Earlier, accumulation of polymer and lack of natural elimination from the cell was a big problem [29]. However, use of natural polymers for nano-formulation has overcome the problem of polymer elimination from the cells with improved bioavailability as well. PLGA is a FDA-approved natural polymer for nanoparticle synthesis with no/low toxicity [30]. Quercetin is generally recognized as safe in humans with no known side effects when taking dosages between 2000 and 5000 mg per day. Additionally, QNP has no known toxicity in normal cells. A nanosuspension of quercetin less than 250 µg/mL concentration is non-cytotoxic to keratinocytes [31]. However, QNPs exhibited a more damaging response to surface morphology in cervical cancer cell lines compared to the bulk quercetin. We also investigated the role of QNPs on cell viability, apoptosis, mitochondrial damage, caspase activation, cell cycle arrest, and tumor inhibition, revealing the underlying molecular mechanisms of cancer progression. Cell viability data showed that QNPs were more toxic to cancer cells than Q, revealing the enhanced therapeutic capability of QNPs. It has been considered that phytochemicals possess the property to control cancer progression via induction of apoptosis, mitochondrial dysfunction, caspase activation, cell cycle arrest, and gene expression alteration [32]. Our results revealed that QNPs showed greater apoptotic effect, mitochondrial dysfunction, caspase activation, and G2 phase cell cycle arrest over bulk quercetin at all the concentrations, demonstrating the potential therapeutic capability of QNPs in cervical and breast cancer. 

More specifically to cancer cell gene expression, quercetin can downregulate Wnt/β-catenin expression in SW480 colon and 4T1 mammary cancer cells [33]. It has been reported to prompt inflammatory and cancer pathways such as EGFR/PI3K/Akt/mTOR, Nrf2/ keap1, MEK/ERK, and STAT3 [9,34]. Previous studies have demonstrated PI3K-mediated apoptosis, proliferation, and cell death in cancer cells [35]. Interestingly, we also noted significant PI3K, p-Akt suppression and FoxO1 activation in QNP-treated cancer cells, suggesting the underlying mechanism to be targeted in cancer reduction. 

To further confirm the therapeutic potency of QNPs as an anticancer agent, an in vivo model, named DMBA-induced mammary adenocarcinoma female SD rats, has been set and tested. QNPs were found to reduce the average number of tumors per animal and extended the tumor latency period (time period between exposure of DMBA and tumor appearance) in rats. Interestingly, a higher dose of QNP was found to be safe and more effective with no specific side effects and toxicity in reducing the DMBA-induced tumor. 

The results indicate that the anti-tumor potential of QNPs opens a new model of a safe and successive plant-derived therapeutic agent. QNPs enhanced the inhibitory role of quercetin in cervical and breast cancer through multiple routes of action, mainly apoptosis induction via PI3K, p-Akt suppression with concomitant caspase, and FoxO1 activation. Subsequently, in vivo results further support the potential capability of QNPs in reducing breast cancer progression. However, there is a need to study the underlying signaling pathways and effect of these QNPs in controlling other cancer-related conditions through clinical studies for successful drug development. 

## 5. Conclusions

This study revealed that PLGA-quercetin nanoparticles have superior effectivity over quercetin on cervical and breast cancer cells via induction of apoptosis, mitochondrial damage, caspase activation, and cell cycle arrest. Specifically, it proves that QNPs induce PI3K, and p-Akt suppression with concomitant caspase and FoxO1 activation. Interestingly, QNPs reduce the tumor volume in DMBA-induced mammary adenocarcinoma female SD rats. It has been proposed that PLGA-QNPs may act as a safe and effective new anticancer agent and the underlying signaling pathway may be a new therapeutic target in cervical and breast cancer treatment. Based on these novel findings, in the future, new anticancer therapies can be developed using different approaches such as a combination of phytochemicals and PLGA via targeting multiple cancer cell lines, exploring different in vivo models to reveal interlinked signaling pathways, and identifying the potential benefits of PLGA-quercetin in different cancer types to achieve targeted drug delivery. 

## Figures and Tables

**Table 1:** Anticancer effect of QNP post-treatment on tumor volume (mm^3^) in breast cancer rats. All the values are expressed as mean ± SEM (*n* = 6).

Group	7 Days (mm^3^)	14 Days (mm^3^)	21 Days (mm^3^)	28 Days (mm^3^)	35 Days (mm^3^)	42 Days (mm^3^)
Control	0	0	0	0	0	0
Vehicle treated with mammary tumor	24.78 ± 2.12	35.79 ± 4.44	43.78 ± 5.44	51.67 ± 6.75	63.67 ± 7.55	73.88 ± 6.55
Q (100 mg/kg, i.p) treated rats	21.66 ± 1.98	27.79 ± 4.03	30.75 ± 5.34	38.89 ± 6.43	45.78 ± 6.57	51.67 ± 7.5
Vehicle control (only PLGA, i.p)	23.66 ± 2.11	37.67 ± 4.096	45.67 ± 5.67	53.78 ± 7.55	64.778 ± 6.45	74.78 ± 6.98
QNPs (128 mg/kg, i.p) treated rats	18.89 ± 1.89	24.78 ± 2.45	29.67 ± 4.03	30.68 ± 4.4	39.78 ± 4.55	40.67 ± 5.42

**Table 2:** Effect of QNP on body weight (g), tumor incidence (mm^3^) and tumor weight on experimental group of animals.

Group	Body Weight (g)	Tumor Incidence (mm^3^)	Tumor Weight (g)
Control	312.44 ± 11.23	0	0
Vehicle (PBST) treated with mammary tumor	216.33 ± 9.34	100%	7.44 ± 1.012
Q (100 mg/kg, i.p) treated rats	234.96 ± 12.67	50%	5.44 ± 0.98
Vehicle control (only PLGA)	214.98 ± 8.94	100%	6.08 ± 0.91
QNPs (128 mg/kg, i.p) treated rats	278.23 ± 14.71	37.8%	4.67 ± 0.73

## Data Availability

The data presented in this study are available on request from the corresponding author. The data are not publicly available due to privacy concerns.
