# Correction: Yadav et al. PLGA-Quercetin Nano-Formulation Inhibits Cancer Progression via Mitochondrial Dependent Caspase-3,7 and Independent FoxO1 Activation with Concomitant PI3K/AKT Suppression. *Pharmaceutics* 2022, *14*, 1326

**DOI:** 10.3390/pharmaceutics16010124

**Published:** 2024-01-18

**Authors:** Neera Yadav, Amit Kumar Tripathi, Amna Parveen, Shama Parveen, Monisha Banerjee

**Affiliations:** 1 College of Pharmacy, Gachon University, #191, Hambakmoeiro, Yeonsu-gu, Incheon 21936, Republic of Korea; yadavneera21@gachon.ac.kr; 2 Molecular and Human Genetics Lab, Department of Zoology, University of Lucknow, Lucknow 226007, India; shamaparveen1192@gmail.com; 3 Electrophysiology Lab, School of Biomedical Engineering, Banaras Hindu University, Varanasi 221005, India; amitbt2008@gmail.com

## Additional Affiliation

In the published publication [1], there was an error regarding the affiliation for Neera Yadav. In addition to affiliation 1, the updated affiliation should include: Molecular and Human Genetics Lab, Department of Zoology, University of Lucknow, Lucknow 226007, India. Neera Yadav did not act as the corresponding author any more.

## Addition of Two Authors

Shama Parveen and Monisha Banerjee (the corresponding author) were not included as authors in the original publication [1]. The corrected Author Contributions statement appears here. The authors state that the scientific conclusions are unaffected. This correction was approved by the Academic Editor. The original publication has also been updated.

Shama Parveen: investigation, formal analysis, methodology;

Monisha Banerjee: project administration, funding acquisition, resources, supervision, writing—review and editing.

## Addition of Acknowledgement

The authors also acknowledge Ratan Singh Ray, CSIR-Indian Institute of Toxicology Research (IITR), Lucknow, India, for the synthesis of nanoparticles; Birbal Sahni Institute of Palaeosciences (BSIP), Lucknow, for the SEM facility; Centre of Excellence, Higher Education Government of Uttar Pradesh for the cell culture facility at the Molecular and Human Genetics Laboratory, Department of Zoology, University of Lucknow, Lucknow-226007, India.

The authors state that the scientific conclusions are unaffected. This correction was approved by the Academic Editor and Editor-in-Chief. The original publication has also been updated.
